# Switching to Bictegravir/Emtricitabine/Tenofovir Alafenamide Fumarate Regimen and Its Effect on Liver Steatosis Assessed by Fibroscan

**DOI:** 10.3390/v17030440

**Published:** 2025-03-19

**Authors:** Marcello Trizzino, Roberta Gaudiano, Dalila Mimì Arena, Luca Pipitò, Claudia Gioè, Antonio Cascio

**Affiliations:** 1 Infectious and Tropical Disease Unit and Sicilian Regional Reference Center for the Fight Against AIDS, AOU Policlinico “P. Giaccone”, 90127 Palermo, Italy; claudia.gioe@policlinico.pa.it; 2 Department of Health Promotion, Mother and Child Care, Internal Medicine and Medical Specialties “G. D’Alessandro”, University of Palermo, 90127 Palermo, Italy; dalilaarena49@gmail.com (D.M.A.); lucapipito@gmail.com (L.P.); 3 Palermo Fast-Track City, Casa dei Diritti, Via Libertà 45, 90143 Palermo, Italy

**Keywords:** bictegravir, NNRTIs, hepatic steatosis, MASLD, liver disease, dyslipidemia

## Abstract

Background: Antiretroviral therapy has transformed HIV infection from a fatal disease to a chronic and manageable condition, but increasing health issues beyond acquired immunodeficiency syndrome, such as metabolic, liver, and cardiovascular diseases, have been observed. Furthermore, the increasing prevalence of HIV strains resistant to older antiretroviral regimens has necessitated a re-evaluation of treatment strategies. Methods: We performed a retrospective, observational study to evaluate the long-term outcomes of an antiretroviral switch from a non-nucleoside reverse transcriptase inhibitor-based to bictegravir-based regimen; this study aimed to assess the impact of this antiretroviral switch on treatment adherence, the safety profile, and virologic outcomes. The secondary objectives were to analyze the changes in lipid, kidney function, liver function, and anthropometric parameters after switching. Results: A total of 25 patients were included in this analysis; virologic suppression was maintained over time, with 100% of patients demonstrating undetectable viral loads at 6, 12, 24, and 36 months. In parallel, a significant increase in CD4+ cell count was observed after switching. No significant differences were observed compared to the previous therapy regarding anthropometric parameters or laboratory parameters. However, a significant reduction in liver steatosis, as assessed by Fibroscan, was observed. Conclusions: bictegravir-based regimens are a valid therapeutic option for people living with HIV, particularly for those with metabolic comorbidities.

## 1. Introduction

The advent of antiretroviral therapy (ART) has revolutionized the management of HIV infection, transforming it from a fatal disease to a chronic and manageable condition [1]. While significant strides have been made in improving the efficacy and tolerability of ART regimens, the emergence of drug resistance and the ongoing pursuit of optimal virologic suppression remain critical challenges. Non-nucleoside reverse transcriptase inhibitors (NNRTIs) have long been a cornerstone of first-line ART due to their potent antiviral activity and favorable side effect profile. However, the increasing prevalence of NNRTI-resistant HIV strains has necessitated a re-evaluation of treatment strategies [2].

The development of drug resistance is a complex process determined by various factors, including the selective pressure exerted by suboptimal drug concentrations, incomplete adherence to therapy, and viral replication capacity [3]. NNRTIs, while highly effective, are associated with a relatively low genetic barrier to resistance. Therefore, even a limited number of genetic changes can be sufficient to confer resistance to these compounds. Once resistance emerges, treatment options become more restricted, and the risk of virologic failure increases [2]. In contrast, integrase strand transfer inhibitors (INSTIs), particularly those with high genetic barriers like bictegravir, are characterized by a more complex resistance profile requiring multiple mutations to develop resistance. This feature makes INSTIs less susceptible to the emergence of drug resistance and offers a more durable treatment option [4,5].

Furthermore, the long-term consequences of suboptimal viral suppression, even at low levels, are not fully understood. Emerging evidence suggests that residual viremia may contribute to ongoing immune activation [6], inflammation, and the development of HIV-related complications, including cardiovascular disease [7], kidney disease [8], and neurocognitive disorders [9]. Achieving and maintaining undetectable viral loads are therefore essential for optimizing long-term health outcomes for people living with HIV (PLWH).

While ART has extended the lifespan of PLWH, it has also led to increased weight gain and health issues beyond acquired immunodeficiency syndrome (AIDS), such as metabolic, liver, and cardiovascular diseases [10]. Metabolic dysfunction-associated steatotic liver disease (MASLD) is currently the most common liver disease among older patients with HIV due to aging, chronic inflammation, and the side effects of ART [11,12].

Non-nucleoside reverse transcriptase inhibitors, especially first-generation ones, have been associated with several mechanisms that can lead to liver damage and are intimately linked to the pathology of MASLD [13]. Furthermore, NNRTIs, while historically a cornerstone of antiretroviral therapy, present certain limitations, including food–drug interactions and the potential for drug resistance [14]. These factors can significantly impact treatment adherence and long-term virologic suppression.

Proactive switching from NNRTI-based to INSTI-based regimens, such as those containing bictegravir, offers several potential advantages. First, it can help to prevent the emergence of drug resistance by reducing the selective pressure on HIV and preserving the susceptibility of the virus to a broader range of antiretroviral agents. Second, it can improve the durability of viral suppression and reduce the risk of treatment failure. Third, it can simplify treatment regimens by reducing the pill burden and improving adherence. Finally, it can provide an opportunity to optimize treatment for individual patients based on factors such as drug resistance, comorbidities, and patient preferences.

This paper argues for the proactive adoption of triple-therapy regimens featuring high-genetic-barrier INSTIs, such as bictegravir, as a more durable and sustainable approach to HIV care. This study aimed to assess the impact of antiretroviral switches from an NNRTI-based regimen to a bictegravir regimen on treatment adherence, the safety profile, and virologic outcomes. We hypothesized that transitioning to a bictegravir-based regimen would improve treatment adherence, reduce adverse events, and maintain or improve virologic suppression.

The secondary objectives were to analyze the changes in lipid profiles (total cholesterol, LDL, HDL, triglycerides), kidney function (eGFR), liver function (GOT, GPT), and anthropometric parameters (weight, waist circumference, body mass index) after switching to bictegravir/emtricitabine/tenofovir alafenamide fumarate (BIC/F/TAF). Additionally, this study aimed to assess changes in the Triglyceride–Glucose (TyG) index, a well-recognized surrogate marker of insulin resistance and metabolic dysfunction-associated fatty liver disease (MAFLD), and the FIB-4 index, a widely used non-invasive marker for liver fibrosis assessment.

## 2. Materials and Methods

### 2.1. Study Design and Setting

This is a real-world, retrospective, observational, monocentric study designed to evaluate the long-term outcomes of the antiretroviral switch from a non-nucleoside reverse transcriptase inhibitor-based regimen to a bictegravir-based regimen in a routine clinical setting. Adults living with HIV were included in the study at the time of switching to BIC/F/TAF from December 2019 to January 2024.

Clinical data collected include sex, age, ethnicity, comorbidities, years of HIV infection, previous antiretroviral therapy regimen, reasons for the switch, and body composition parameters such as weight, waist circumference, body mass index, and Fibroscan results (collected at baseline and 6, 12, 24, and 36 months after the switch).

Laboratory data including HIV-RNA, CD4+ cell count, and biochemical data (total cholesterol, LDL, HDL, triglycerides, eGFR, GOT, GPT, TyG index) were collected at baseline and 6, 12, 24, and 36 months after the switch.

### 2.2. Inclusion and Non-Inclusion Criteria

Inclusion criteria: PLWH > 18 years old; switched from NNRTI-based regimen to BIC/F/TAF for any reason. Non-inclusion criteria: virologic failure of the previous regimen (defined as two consecutive HIV-1 RNA measurements > 200 copies/mL); pregnancy; presence of HIV genotypes with resistance mutations to bictegravir; patients who had experienced adverse effects related to bictegravir.

### 2.3. Statistics

Statistical analyses were performed using SPSS 19.0. Parametric tests were used for normally distributed data, while non-parametric tests were employed for data that did not meet the assumptions of normality. For statistical analysis, we considered a 5% significance level.

## 3. Results

This analysis included 25 patients who switched from a non-nucleoside reverse transcriptase inhibitor-based regimen to bictegravir/emtricitabine/tenofovir alafenamide in routine clinical practice from December 2019 to January 2024. Their baseline demographic and clinical characteristics are presented in Table 1.

Most patients were male (84%) and Caucasian (92%), with a mean history of HIV infection of 18.24 years (3–33). Almost the entire population (96%) had a single-tablet regimen (STR) before the switch, and the median number of previous antiretroviral therapy regimens was 2 (1–7). Only 4% of the population had a history of AIDS-defining opportunistic illnesses. Among the included participants, one did not show virological suppression (HIV-1 RNA ≥ 50 copies/mL) at baseline.

Of the patients, 80% had at least one comorbidity, and 28% presented two or more medical conditions. The most common associated diseases were dyslipidemia (48%), hypertension (32%), osteoporosis (16%), neoplasm (16%), diabetes (8%), chronic kidney failure (8%), obesity (8%), and ischemic cardiac disease (4%).

Before the switch, the majority of patients (84%) were on rilpivirine/emtricitabine/tenofovir alafenamide; the other regimens were efavirenz/emtricitabine/tenofovir disoproxil fumarate (12%) and doravirine + dolutegravir (4%).

The primary reasons for switching to BIC/F/TAF were drug–drug interactions (32%), simplification (30%), a proactive choice by the healthcare provider and patient (20%), elevated metabolic and cardiovascular risks (20%), and the side effects of the previous antiretroviral therapy (8%).

After the switch to BIC/F/TAF, patients were followed for a median of 561 days (IQR 547–1668). During the observation period, only one patient interrupted BIC/F/TAF treatment due to simplification to a dual regimen.

**Figure 1:**
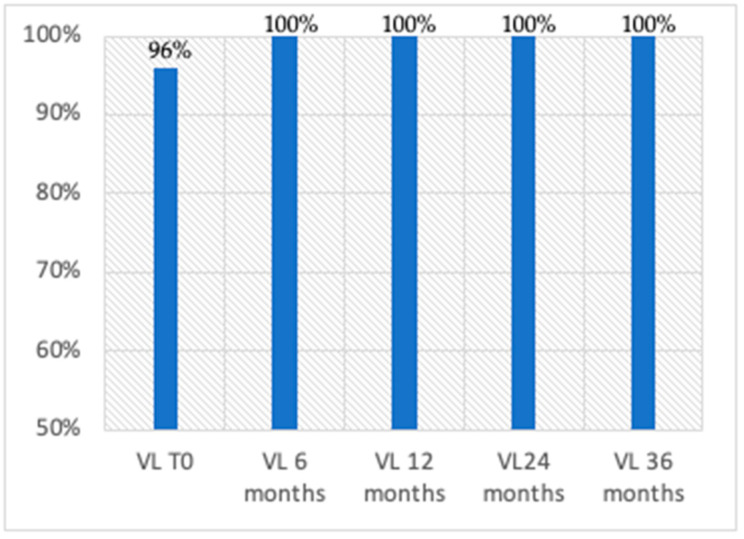
Percentage of patients with HIV-RNA < 50.0 copies/mL.

**Figure 2:**
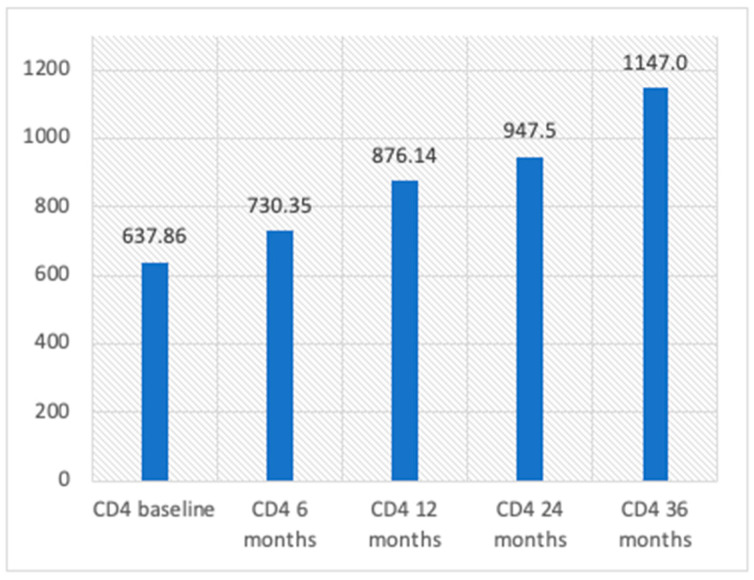
CD4 count during follow-up.

Virologic suppression was maintained over time, with 100% of patients demonstrating undetectable viral loads at 6, 12, 24, and 36 months. Furthermore, a significant increase in CD4+ cell count was observed, rising from a baseline of 637 cells/mm^3^ to over 1100 cells/mm^3^ after 26 months of treatment with BIC/F/TAF (Figure 1 and Figure 2).

Regarding the safety of the new regimen, no significant differences were observed compared to the previous therapy regarding anthropometric parameters (weight, waist circumference, body mass index) or laboratory parameters. However, a significant reduction in liver steatosis, as assessed by Fibroscan, was observed (Table 2).

## 4. Discussion

The efficacy of bictegravir/emtricitabine/tenofovir alafenamide in achieving and maintaining virologic suppression has been consistently demonstrated in both randomized controlled trials and real-world studies [15,16]. Numerous large-scale clinical trials have shown that BIC/F/TAF rapidly and durably suppresses HIV-1 RNA to undetectable levels in treatment-naïve and experienced patients. These findings have been corroborated by real-world evidence from observational studies, which have documented high rates of virologic suppression and excellent tolerability in diverse patient populations [15,16]. Our study further supports these findings, as we observed a high rate of virologic suppression among participants treated with BIC/F/TAF, comparable to that reported in pivotal trials. These results reinforce the robust efficacy of BIC/F/TAF in suppressing HIV replication and highlight its potential as a first-line and switch therapy for people living with HIV [15,16,17].

Multiple clinical and practical considerations influenced our cohort’s decision to switch to bictegravir/emtricitabine/tenofovir alafenamide. The primary motivations for switching included drug–drug interactions (32%), regimen simplification (30%), a proactive choice by the healthcare provider and patient (20%), elevated metabolic and cardiovascular risks (20%), and the side effects of the previous antiretroviral therapy (8%). These factors reflect the evolving landscape of HIV management, where optimizing long-term tolerability, safety, and adherence is prioritized alongside virologic suppression.

Drug–drug interactions were a significant driver for switching to BIC/F/TAF, particularly among patients receiving regimens that included rilpivirine or efavirenz. Rilpivirine, a non-nucleoside reverse transcriptase inhibitor, has well-documented interactions with proton pump inhibitors and antiepileptic drugs [18], limiting treatment options for patients with comorbid conditions. Similarly, efavirenz is associated with cytochrome P450 enzyme induction, which can impact the metabolism of various concomitant medications, including anticoagulants, statins, and psychotropic drugs [19]. The transition to BIC/F/TAF in these cases was motivated by its minimal drug–drug interaction profile, offering greater flexibility for managing polypharmacy in aging patients with HIV.

Regimen simplification accounted for 30% of switches, underscoring the preference for treatment strategies that enhance convenience and adherence. Although many patients were already on single-tablet regimens, subtle differences in tolerability and pharmacokinetics influenced their decision to transition to BIC/F/TAF. In particular, some individuals experienced difficulties related to food requirements [14] with rilpivirine-based therapies. By eliminating these restrictions, BIC/F/TAF provided an optimized treatment option that maintained efficacy while improving the overall patient experience.

A proactive choice by healthcare providers and patients played a role in 20% of switches. Given the increasing recognition of long-term ART-related complications, many clinicians and patients opted for a regimen with a more favorable safety profile. The decision to pre-emptively switch to bictegravir/emtricitabine/tenofovir alafenamide was guided by emerging evidence on bictegravir’s high genetic barrier to resistance and its well-tolerated nature. Additionally, as guidelines continue to emphasize the importance of patient-centered care, shared decision-making between clinicians and individuals living with HIV is becoming a key component in treatment optimization.

Metabolic and cardiovascular risks were another major factor influencing antiretroviral therapy switches, particularly for patients on efavirenz- or tenofovir disoproxil fumarate (TDF)-based regimens. Efavirenz has been associated with dyslipidemia [20] and neuropsychiatric side effects [21], while TDF is linked to reductions in bone mineral density [22] and potential renal toxicity [23]. The transition to BIC/F/TAF, which contains tenofovir alafenamide fumarate (TAF), was made with the aim of mitigating these concerns by offering a regimen with improved lipid and renal safety profiles. For patients with pre-existing metabolic conditions or an elevated risk of cardiovascular disease, switching to BIC/F/TAF was a proactive step to minimize long-term complications.

Lastly, 8% of patients switched due to persistent but non-severe side effects of their previous antiretroviral therapy. Symptoms such as gastrointestinal discomfort, insomnia, or neurocognitive disturbances can significantly impact quality of life and adherence, even if they do not necessitate immediate regimen discontinuation. For these individuals, bictegravir/emtricitabine/tenofovir alafenamide provided an opportunity to maintain viral suppression while potentially improving tolerability and overall treatment satisfaction.

Although the five patients who switched to bictegravir/emtricitabine/tenofovir alafenamide for “simplification” were already receiving a single-tablet regimen, their decision was driven by specific clinical considerations beyond mere regimen consolidation. Some patients experienced low-grade but persistent side effects with their previous STR, including gastrointestinal discomfort, sleep disturbances, or neurocognitive symptoms, which, while not severe enough to warrant immediate discontinuation, contributed to reduced overall treatment satisfaction and adherence.

Furthermore, recent evidence suggests that certain single-tablet regimens may have pharmacokinetic profiles that, despite providing effective viral suppression, can lead to mild systemic intolerance in susceptible individuals [24,25]. The transition to BIC/F/TAF in these cases was made with the aim of improving tolerability while maintaining a simplified regimen. Additionally, given BIC/F/TAF’s favorable drug–drug interaction profile, some patients with polypharmacy concerns benefited from a regimen that minimized the risk of metabolic or renal complications associated with previous treatments.

These findings highlight the multifaceted considerations involved in antiretroviral therapy regimen selection, emphasizing that while virologic efficacy remains paramount, treatment decisions should also account for drug–drug interactions, metabolic safety, the side effect burden, and patient-reported outcomes to optimize long-term success. An individualized approach to ART is crucial, ensuring that regimen choices are not dictated solely by the number of tablets but also by broader factors influencing adherence, quality of life, and long-term metabolic health. Future research should continue exploring the impact of ART transitions, delving beyond virologic efficacy to refine strategies that enhance patient outcomes and sustain treatment success.

A cornerstone of successful antiretroviral therapy is the ability of a regimen to suppress viral replication upon reintroduction into a treatment setting. This characteristic, known as forgiveness, is crucial for maintaining long-term virologic control and improving patient outcomes. Regimens with high forgiveness rates allow for occasional treatment interruptions or adherence lapses without leading to rapid viral rebound, reducing the risk of treatment failure and the emergence of drug resistance [26,27].

Bictegravir/emtricitabine/tenofovir alafenamide has consistently demonstrated exceptional forgiveness properties in clinical and real-world trials. Multiple studies have shown that patients who experienced temporary interruptions in their BIC/F/TAF regimen were able to regain virologic suppression rapidly upon restarting therapy. This high level of forgiveness can be attributed to several factors, including the potent antiviral activity of bictegravir, the long half-lives of the components, and the favorable pharmacokinetic properties of the combination [26,27].

The forgiveness of BIC/F/TAF offers several advantages for patients living with HIV. It provides a safety net for individuals who may experience challenges with adherence, reducing the anxiety associated with missed doses. Furthermore, it allows for greater flexibility in treatment management, enabling clinicians to adjust therapy as needed to address individual patient needs. In conclusion, the high level of forgiveness exhibited by BIC/F/TAF is a significant advantage of this regimen and contributes to its overall efficacy and tolerability [26,27].

One of the most significant challenges in the management of HIV is ensuring long-term adherence to antiretroviral therapy. Adherence is crucial for achieving viral suppression, preventing disease progression, and reducing the risk of drug resistance. Several factors influence adherence, including regimen complexity, side effects, and patient-related factors. Single-tablet regimens have emerged as a promising approach to improve adherence by simplifying dosing and reducing the pill burden [28].

Bictegravir, an integrase strand transfer inhibitor, has demonstrated exceptional efficacy and safety in clinical trials and real-world settings [29]. When combined with emtricitabine and tenofovir alafenamide in a fixed-dose combination tablet, bictegravir offers a potent and well-tolerated treatment option for people living with HIV [30]. The single-tablet regimen formulation has significantly contributed to the high levels of adherence observed with this regimen [31].

Several studies have consistently shown that patients receiving bictegravir-based STRs have high rates of adherence [32]. The ease of taking a single pill once daily has been a significant factor in improving adherence. Additionally, the favorable safety profile of bictegravir has contributed to its high tolerability [16]. In clinical trials, bictegravir has been associated with a low incidence of adverse events, particularly those that could lead to treatment discontinuation. Real-world studies have further confirmed these findings, demonstrating that bictegravir-based regimens are well tolerated in diverse patient populations [33].

The high level of adherence observed with bictegravir-based regimens has several important implications for clinical practice. First, it suggests that these regimens may be particularly suitable for patients who have historically struggled with adherence to more complex antiretroviral therapy regimens. Second, high adherence rates are associated with better clinical outcomes, including sustained viral suppression and improved quality of life. Finally, the high tolerability of bictegravir-based regimens may reduce the need for treatment modifications and enhance patient satisfaction.

The results of this study provide compelling evidence supporting the efficacy and safety of switching to bictegravir-based regimens for PLWH. Specifically, the data demonstrate that this therapeutic strategy can maintain high-level virologic suppression over the long term, confirming its position as a viable alternative to traditional NNRTI-based regimens. The proactive adoption of triple-therapy regimens featuring high-genetic-barrier INSTIs, such as bictegravir, represents a significant advance in the management of HIV infection. By providing a more durable and sustainable treatment option, these regimens can help to improve long-term health outcomes for people living with HIV and reduce the burden of HIV on healthcare systems.

Beyond bictegravir’s excellent virologic efficacy profile, this study highlighted a noteworthy reduction in hepatic steatosis among patients who switched to this medication. This finding is clinically significant given the high prevalence of metabolic comorbidities, including diabetes mellitus and dyslipidemia, in PLWH. The reduction in hepatic steatosis, as assessed by the controlled attenuation parameter (CAP), is of paramount importance in the HIV population, which is aging and increasingly susceptible to age-related phenomena such as senescence. The chronic inflammatory state associated with HIV infection exacerbates these age-related changes, rendering hepatic steatosis a significant risk factor for the development of more severe liver diseases [34].

In addition to standard clinical assessments, all patients underwent a Fibroscan examination to evaluate the liver stiffness and CAP to assess hepatic steatosis. Fibroscan is a non-invasive method that uses transient elastography to measure liver stiffness, a surrogate marker of liver fibrosis, while the CAP quantitatively assesses liver fat content [35].

Hepatic steatosis is a common complication of these conditions and can progress to more severe forms of liver disease, increasing the risk of cirrhosis and hepatocellular carcinoma [36]. Furthermore, MASLD and liver fibrosis increase cardiovascular risk in HIV [37].

By quantitatively measuring liver fibrosis and steatosis, Fibroscan can provide an effective risk stratification of PLWH, guide treatment decisions, and improve overall outcomes [35]. Using this diagnostic device, we aimed to comprehensively investigate the potential impact of bictegravir-based regimens on liver health, given the high prevalence of metabolic comorbidities among PLWH and the strong association between these comorbidities and liver disease [34].

In addition to the CAP and Fibroscan, the FIB-4 index should be incorporated into routine assessments of PLWH to evaluate liver fibrosis. The FIB-4 index is a simple and non-invasive biomarker that uses age, AST, ALT, and platelet count to estimate the risk of significant liver fibrosis. Studies have demonstrated its reliability in assessing liver fibrosis in patients with HIV, reinforcing its role in guiding clinical management and early intervention strategies [38,39,40]. Implementing the FIB-4 index in routine care could enhance the early detection of liver complications and optimize long-term health outcomes for PLWH.

A comprehensive evaluation of PLWH should also include the assessment of surrogate markers of insulin resistance, such as the Triglyceride–Glucose (TyG) index. The TyG index, which is easily calculated using fasting triglyceride and glucose levels, is an established marker of insulin resistance, similar to the HOMA-IR index. Including this parameter in clinical assessments could provide valuable insights into metabolic health and cardiovascular risk in PLWH. Recent studies have highlighted the importance of the TyG index in identifying patients at higher risk of metabolic complications, further supporting its role in routine evaluations [41,42].

Although no significant differences were observed compared to the previous therapy regarding the FIB-4 index and TyG index, a significant reduction in liver steatosis assessed by Fibroscan was observed. This finding suggests that while specific metabolic and fibrotic markers remained stable following the transition to the bictegravir-based regimen, liver fat content experienced a measurable decline. The Fibroscan-based reduction in hepatic steatosis is particularly relevant given the increasing prevalence of MASLD in PLWH [12]. The absence of significant changes in the FIB-4 and TyG indices could indicate that while hepatic steatosis was positively affected, more profound alterations in fibrosis progression or insulin resistance did not manifest within this study’s timeframe. However, it is important to interpret these findings within the context of the study’s duration, the sample size, and the biological mechanisms underlying these indices.

The FIB-4 index, a widely used non-invasive marker for liver fibrosis assessment, integrates age, AST, ALT, and platelet count to estimate the degree of hepatic fibrosis. Despite its utility in identifying individuals at risk of advanced liver disease, FIB-4 may be less sensitive to short-term fluctuations in liver health, particularly in therapeutic interventions aimed at improving hepatic metabolism rather than directly targeting fibrosis. Given that fibrosis progression often occurs over a prolonged period, it is plausible that a longer follow-up period would be required to detect meaningful changes in the FIB-4 index. Additionally, since Fibroscan measures liver stiffness and fat content independently of the biochemical parameters considered in the FIB-4 score, the observed reduction in steatosis may not have been immediately reflected in FIB-4 values.

Similarly, the TyG index, a surrogate marker of insulin resistance, did not exhibit significant changes following the therapy switch. This observation suggests that while the new regimen may have improved hepatic lipid accumulation, broader metabolic parameters related to glucose metabolism and insulin sensitivity were not significantly altered within the study period.

The significant reduction in liver steatosis observed in this study highlights the potential benefits of bictegravir-based regimens beyond virologic suppression. Given the well-documented impact of chronic HIV infection and antiretroviral therapy on metabolic health [43], the observed improvement in liver fat content suggests that the new regimen may offer advantages in managing MASLD among PLWH. While the exact mechanisms remain to be elucidated, potential contributing factors include improved mitochondrial function, reduced hepatic inflammation, and favorable shifts in lipid metabolism.

The findings of our study contrast with those reported by Calza et al. [42], which suggested an improvement in metabolic parameters following the switch from an INSTI-based regimen to an NNRTI-based regimen. Several factors may account for these discrepancies. First, differences in patient populations may have influenced outcomes, as our cohort primarily included individuals with pre-existing metabolic comorbidities. In contrast, the study by Calza et al. may have assessed a more metabolically stable population. Second, variations in methodology, including the use of Fibroscan in our study versus alternative metabolic assessment tools in Calza et al., may have led to differing conclusions regarding liver steatosis. Third, the duration of follow-up in our study extended up to 36 months, allowing for a longer observational period to detect changes in hepatic steatosis. In contrast, the follow-up duration in Calza et al. may have been shorter, potentially limiting the ability to capture long-term metabolic effects. The potential confounding effects of concomitant medications, dietary habits, and lifestyle factors may have contributed to the observed differences. Given these factors, further prospective, multicenter studies are warranted to better elucidate the impact of antiretroviral therapy switching strategies on metabolic health and liver function in PLWH.

Future research should focus on longitudinal studies with extended follow-up periods and larger cohorts to determine whether reductions in hepatic steatosis translate into long-term improvements in liver fibrosis and overall metabolic health.

A potential limitation of this study is the exclusive use of Fibroscan and the FIB-4 index to assess hepatic steatosis. While Fibroscan is a widely validated, non-invasive tool for evaluating liver stiffness and fat accumulation, its findings could have been further corroborated using additional imaging or histological techniques. Magnetic resonance imaging with the proton density fat fraction is considered a highly sensitive method for quantifying liver fat content; however, its routine clinical application is limited by its high costs, restricted availability, and logistical constraints, making it impractical for large-scale or real-world studies [44].

Similarly, liver biopsy remains the gold standard for diagnosing and staging hepatic steatosis and fibrosis. Nevertheless, its invasive nature, associated procedural risks (e.g., bleeding, infection), and potential sampling variability render it unsuitable for routine monitoring, particularly in asymptomatic or stable patients [45,46,47]. Given these considerations, Fibroscan has emerged as the preferred non-invasive alternative, offering a reliable, reproducible, and patient-friendly assessment of liver health.

Our findings suggest that clinicians should take a more individualized approach to antiretroviral therapy selection, considering viral suppression and metabolic outcomes when choosing treatment regimens. This study highlights the importance of integrating metabolic health assessments into routine HIV care, which may include regular Fibroscan evaluations, metabolic profiling, and the monitoring of cardiovascular risk factors. By incorporating these additional evaluations, clinicians can identify patients who may benefit most from switching to regimens such as bictegravir/emtricitabine/tenofovir alafenamide.

Furthermore, the demonstrated high tolerability and safety profile of BIC/F/TAF reinforce its role as a viable therapeutic option in routine clinical practice. The simplification of treatment regimens remains a critical objective in HIV management, and switching to a single-tablet regimen like BIC/F/TAF can enhance patient adherence, minimize the pill burden, and reduce the likelihood of drug–drug interactions. These advantages can contribute to long-term treatment success, ensuring sustained virologic suppression and improved quality of life for PLWH.

The potential impact of ART regimens on hepatic steatosis also underscores the need for interdisciplinary collaboration between infectious disease specialists, hepatologists, and metabolic disease experts. A multidisciplinary approach to HIV care could help refine treatment strategies by incorporating liver health as a central component of antiretroviral therapy optimization. Future studies should continue exploring the broader metabolic effects of BIC/F/TAF in more extensive and diverse cohorts, potentially leading to refined treatment guidelines that integrate metabolic considerations into ART selection. Additionally, prospective studies evaluating long-term outcomes could provide valuable insights into the durability of these metabolic benefits and their impact on overall patient health.

This study provides innovative insights into the potential benefits of switching to bictegravir/emtricitabine/tenofovir alafenamide, particularly in the context of hepatic steatosis management in PLWH. While previous studies have extensively documented the virologic efficacy, safety, and tolerability of BIC/F/TAF, our research adds valuable evidence regarding its metabolic impact, specifically on liver health.

1. Reduction in hepatic steatosis: One of the most relevant findings of our study is the observed reduction in hepatic steatosis as assessed by Fibroscan. This finding suggests a potential metabolic advantage of BIC/F/TAF, which could be crucial for patients with HIV at risk of MASLD. Given the increasing burden of MASLD among PLWH due to aging, chronic inflammation, and antiretroviral therapy-related metabolic effects, our results highlight the need for further investigation into the role of ART regimens in liver health.
2. Re-evaluation of the role of INSTIs in metabolism: Contrary to concerns raised by some studies regarding the potential adverse metabolic effects of INSTIs, our findings suggest a beneficial or neutral impact of BIC/F/TAF on liver steatosis. This discrepancy underlines the importance of patient-specific factors and the need for a more nuanced understanding of antiretroviral therapy-related metabolic effects.
3. Real-world clinical relevance: Unlike controlled clinical trials, our study reflects real-world clinical practice, making the findings highly relevant for routine HIV management. By providing data from a real-life cohort, we contribute to the growing body of evidence supporting the long-term metabolic safety of BIC/F/TAF. The observed improvement in hepatic steatosis following BIC/F/TAF initiation suggests its potential benefit for patients with coexisting metabolic conditions. Additionally, the high adherence and tolerability in our cohort highlight the feasibility of this regimen in routine HIV care. Future studies should incorporate larger, multicenter cohorts with extended follow-up periods to validate these findings and further explore long-term metabolic outcomes.
4. Comparison with larger studies: To further substantiate our findings, we will consider comparing our results with those obtained in larger cohorts or meta-analyses evaluating the metabolic effects of BIC/F/TAF. This comparison would allow us to assess the consistency of our observations and determine whether the metabolic benefits we identified are generalizable across different populations. Larger studies with more extensive datasets may provide additional statistical power to validate our findings and minimize the potential impact of selection bias inherent in smaller, single-center studies. Evaluating our results alongside broader clinical evidence will help establish the reliability and clinical significance of BIC/F/TAF in improving hepatic and metabolic parameters in PLWH. Additionally, meta-analytical approaches integrating data from multiple studies could provide a more comprehensive understanding of the treatment’s impact across diverse patient groups and clinical settings.
5. Implications for future research: Our results open new avenues for research into the interplay between HIV, antiretroviral therapy, and metabolic health. Future studies should aim to confirm our findings in larger, multicenter cohorts and investigate the underlying mechanisms driving the observed improvements in liver steatosis.

By emphasizing these novel aspects, this study strengthens the discussion on the benefits of switching to BIC/F/TAF beyond virologic control, particularly in metabolic and hepatic health. These findings could influence future treatment guidelines and optimize antiretroviral therapy choices for patients with metabolic comorbidities.

However, this study has several limitations to consider when interpreting the results.

1. Sample size: The number of patients included in this study is relatively small (n = 25), which may limit the generalizability of the findings. A more extensive study could provide more robust data and statistical power to confirm the reported observations.
2. Lack of additional metabolic parameters: Data on insulin resistance and fasting glucose levels were not collected despite their strong association with hepatic steatosis and metabolic syndrome. Including these parameters in future analyses could provide a more comprehensive assessment of liver metabolism in patients undergoing bictegravir/emtricitabine/tenofovir alafenamide therapy.
3. Comparison with previous studies: Our findings on the reduction in hepatic steatosis after switching to BIC/F/TAF contrast with some recent studies suggesting a worsening of metabolic parameters after switching from an INSTI to an NNRTI. This discrepancy may be due to differences in the study population, follow-up duration, or hepatic steatosis assessment methods. Future research should explore these differences more thoroughly.
4. Reason for therapy switch: Therapy simplification was cited as a reason for switching in five patients, despite most previous regimens already being single-tablet formulations. This aspect requires further clarification for a better understanding of the underlying clinical rationale.
5. Exclusive use of Fibroscan for steatosis assessment: Although Fibroscan is a validated tool for measuring hepatic steatosis, its use as the sole method may be a limitation. Integrating other diagnostic techniques, such as proton magnetic resonance spectroscopy or liver biopsy, could provide more accurate data on steatosis progression in patients with HIV.
6. Observational nature of the study: Given that this was a retrospective, single-center study, our findings may be influenced by selection bias and unaccounted confounding factors. Prospective, multicenter studies could provide more robust data to confirm our observations.

Despite these limitations, our study provides relevant insights into the management of hepatic steatosis in patients with HIV undergoing bictegravir/emtricitabine/tenofovir alafenamide therapy. However, further research is needed to confirm our findings and explore the underlying mechanisms of the observed metabolic changes.

## 5. Conclusions

The presented data support the use of bictegravir-based regimens as a valid therapeutic option for PLWH, particularly for those with metabolic comorbidities. The ability to maintain high-level virologic suppression and improve patients’ metabolic profiles makes this class of drugs a valuable tool in the long-term management of HIV infection. However, further studies are needed to evaluate the long-term impact of bictegravir on liver disease progression and to identify the subgroups of patients who may benefit most from this therapy.

## Figures and Tables

**Table 1:** Baseline demographic and clinical characteristics.

	BIC/F/TAF (*n* = 25)
Male	21 (84%)
Female	4 (16%)
Years of HIV infection	18.24 (3–33)
CD4+ before switch	637.86 (173–912)
Previous regimens	
RPV/TAF/FTC	21 (84%)
EFV/TDF/FTC	3 (12%)
DTG + DOR	1 (4%)
Days in BIC/F/TAF	829 (258–1760)
Reasons for the switch	
Drug interactions	8 (32%)
Simplification	5 (20%)
Previous regime toxicity	2 (8%)
Proactive switch	2 (8%)
High metabolic/CV risk	5 (20%)
≥2 reasons for switching	3 (12%)
Comorbidities	
≥2 comorbidities	10 (40%)
≥3 comorbidities	6 (24%)
Multimorbidity	7 (28%)
Polypharmacy	6 (36%)
Cigarette smoking	11 (44%)
≥3 non-cART medications	8 (32%)

**Table 2:** Anthropometric, laboratory, and controlled attenuation parameters during follow-up.

Parameters	Values	*p*
Weight T0–Weight 6 months	76.25–76.75	0.502
Weight T0–Weight 12 months	76.00–75.50	0.874
Waist circumference T0–Waist circumference 6 months	99.25–101.00	0.144
BMI T0–BMI 6 months	25.67–25.83	0.611
BMI T0–BMI 12 months	26.00–25.50	0.500
Cholesterol T T0–Cholesterol T 6 months	166.47–169.53	0.664
Cholesterol T T0–Cholesterol T 12 months	144.57–159.57	0.189
Cholesterol T T0–Cholesterol T 24 months	140.17–149.50	0.380
Cholesterol T T0–Cholesterol T 36 months	157.50–191.00	0.104
Cholesterol LDL T0–Cholesterol LDL 6 months	90.82–100.18	0.112
Cholesterol LDL T0–Cholesterol LDL 12 months	74.33–83.67	0.456
Cholesterol LDL T0–Cholesterol LDL 24 months	59.00–70.00	0.563
Cholesterol LDL T0–Cholesterol LDL 36 months	75.50–81.00	0.914
Triglycerides T0–Triglycerides 6 months	123.11–112.16	0.510
Triglycerides T0–Triglycerides 12 months	92.43–103.57	0.423
Triglycerides T0–Triglycerides 24 months	122.33–91.83	0.489
Triglycerides T0–Triglycerides 36 months	72.50–11.50	0.352
eGFR T0–eGFR 6 months	96.17–87.22	0.180
eGFR T0–eGFR 12 months	79.43–71.86	0.075
eGFR T0–eGFR 24 months	95.83–91.00	0.264
eGFR T0–eGFR 36 months	82.00–62.00	0.344
AST T0–AST 6 months	26.95–23.90	0.312
AST T0–AST 12 months	32.00–24.50	0.484
AST T0–AST 24 months	35.86–27.71	0.191
AST T0–AST 36 months	49.00–25.00	0.274
CAP T0–CAP T1 (>12 months)	308.00–225.50	0.027
Tyg Index T0–Tyg Index 6 months	4.63–4.55	0.297
Tyg Index T0–Tyg Index 12 months	4.47–4.47	0.997
Tyg Index T0–Tyg Index 24 months	4.56–4.43	0.442
Fib-4 T0–Fib-4 6 months	1.38–1.29	0.577
Fib-4 T0–Fib-4 12 months	1.83–1.40	0.303
Fib-4 T0–Fib-4 24 months	1.62–1.26	0.365
Fib-4 T0–Fib-4 36 months	2.24–1.26	0.428

## Data Availability

The original contributions presented in this study are included in the article. Further inquiries can be directed to the corresponding authors.

## Abbreviations

The following abbreviations are used in this manuscript:

AIDS	Acquired Immunodeficiency Syndrome
ART	Antiretroviral Therapy
BIC/F/TAF	Bictegravir/Emtricitabine/Tenofovir alafenamide fumarate
CAP	Controlled Attenuation Parameter
INSTI	Integrase Strand Transfer Inhibitor
MASLD	Metabolic Dysfunction-Associated Steatotic Liver Disease
NNRTI	Non-Nucleoside Reverse Transcriptase Inhibitor
PLWH	People Living With HIV
STR	Single-Tablet Regimen
TAF	Tenofovir Alafenamide Fumarate
TDF	Tenofovir Disoproxil Fumarate
